# Improving the reaction mix of a *Pichia pastoris* cell-free system using a design of experiments approach to minimise experimental effort

**DOI:** 10.1016/j.synbio.2020.06.003

**Published:** 2020-06-23

**Authors:** Alex J. Spice, Rochelle Aw, Daniel G. Bracewell, Karen M. Polizzi

**Affiliations:** a Department of Chemical Engineering, Imperial College London, London, UK; b Imperial College Centre for Synthetic Biology, Imperial College London, UK; c Department of Biochemical Engineering, University College London, London, UK

**Keywords:** Cell-free protein synthesis, *Pichia pastoris*, Synthetic biology, Design of experiments (DOE), AB, Albumin Blue, CFPS, cell-free protein synthesis, DOE, design of Experiments, DSD, definitive screening design, HSA, human serum albumin, IRES, internal ribosome entry site, CHO, Chinese hamster ovary cells, WGE, wheat-germ etract, RRL, rabbit reticulocyte lysate, VLP, virus-like particles

## Abstract

A renaissance in cell-free protein synthesis (CFPS) is underway, enabled by the acceleration and adoption of synthetic biology methods. CFPS has emerged as a powerful platform technology for synthetic gene network design, biosensing and on-demand biomanufacturing. Whilst primarily of bacterial origin, cell-free extracts derived from a variety of host organisms have been explored, aiming to capitalise on cellular diversity and the advantageous properties associated with those organisms. However, cell-free extracts produced from eukaryotes are often overlooked due to their relatively low yields, despite the potential for improved protein folding and posttranslational modifications. Here we describe further development of a *Pichia pastoris* cell-free platform, a widely used expression host in both academia and the biopharmaceutical industry. Using a minimised Design of Experiments (DOE) approach, we were able to increase the productivity of the system by improving the composition of the complex reaction mixture. This was achieved in a minimal number of experimental runs, within the constraints of the design and without the need for liquid-handling robots. In doing so, we were able to estimate the main effects impacting productivity in the system and increased the protein synthesis of firefly luciferase and the biopharmaceutical HSA by 4.8-fold and 3.5-fold, respectively. This study highlights the *P. pastoris-*based cell-free system as a highly productive eukaryotic platform and displays the value of minimised DOE designs.

## 1 Introduction

The ability to synthesize proteins without the complete machinery of living cells was first demonstrated in the early sixties [1]. In the years since this pioneering discovery, vast progress has been made in improving cell-free protein synthesis (CFPS) systems and the scope of their applications. Compared to protein expression *in vivo*, CFPS offers a number of advantages, such as the ability to rapidly synthesize proteins on-demand, circumvention of laborious cloning and transformation procedures, and direct control and monitoring of the reaction environment in real-time [2]. Additionally, the open nature of the reaction environment allows unparalleled accessibility, enabling cell-free platforms to be modulated with an ease far greater than *in vivo* systems. Cell-free systems are therefore highly amenable to high-throughput screening and rapid prototyping for characterisation and optimisation purposes. Due to these advantageous properties, cell-free systems can be employed in a diverse range of applications. Such applications include the design of *de novo* metabolic pathways [3], personalised medicine and decentralised manufacturing [4] and increasingly as biosensors [5]. CFPS has also shown to be highly effective in the production of difficult-to-express proteins, such as those toxic to the cell *in vivo* [6]*.*

Today there are multiple sources of cell-free extracts, however *Escherichia coli* lysates are still the most widely used, primarily due to their low-cost, high yields and demonstrable scalability [7]. The other most commonly used systems, and those which are commercially available, are from HeLa cells [8], wheat-germ extract (WGE) [9,10], rabbit reticulocyte lysate (RRL) [11] and insect (Sf9) cells [12]. In recent years the variety of developed CFPS extracts has been expanded, now including HEK293 [13], Chinese hamster ovary cells (CHO) [14], *Saccharomyces cerevisiae* [15], *BY-2* tobacco cells [16], *Streptomyces venezuelae* [17], *Bacillus megaterium* [18], *Vibrio natriegens* [19,20] and *Pichia pastoris* (syn. *Komagataella phaffi*) [21].

We have previously developed a coupled CFPS system based on *P. pastoris* lysates [21] and demonstrated its capability to produce virus-like particles [22]. Initial attempts to improve the productivity of the *P. pastoris* cell-free platform included maximising the translational capacity of the system by engineering a ribosome biogenesis modified strain FHL1, from which the lysates are derived. Further improvements to the system were achieved by modifying the CFPS expression vectors to include IRES elements in order to improve translation initiation in a cap-independent manner thereby boosting productivity [21].

In this study, we have turned our attention towards improvement of the reaction mix composition in order to further enhance the productivity of the *P. pastoris* cell-free system. Despite cell-free systems being relatively simple compared to whole-cell expression systems, they have historically always had complex and expensive reaction mixtures with a network of interacting factors, both within the reaction mix and the lysate itself [23]. Attempting to modify the reaction mix using a one-factor-at-a-time approach is ineffective at optimising protein synthesis (yield), due to the presence of strong nonlinear interactions between factors [23]. In the past, design of experiments (DOE) approaches have proven to be successful for characterisation and optimisation of cell-free systems when used in conjunction with high-throughput methodologies, such as automation [[23], [24], [25], [26], [27], [28], [29]]. Using this approach, many different systems have been improved, both in terms of the overall robustness and productivity. DOE has been successfully employed in the optimisation of the reaction mix composition of CFPS platforms derived from insect cells [24], tobacco BY-2 cell lysates (BYLs) [30] and *E. coli* [23,25,31,32]. One of the main challenges in attempting similar approaches using the *P. pastoris* system is the inability to produce a reporter protein which can be directly measured in the reaction environment, such as green-fluorescent protein (GFP). This was similarly reported in the *Saccharomyces cerevisiae* cell-free platform [15], where a luciferase reporter assay was adopted instead. Unfortunately, using a luciferase-based assay increases sampling handling requirements in order to quantify the synthesised protein present in the reaction at any given time. As a result of this we have utilised a minimised DOE approach, that can be implemented without the use of automation, to improve the reaction mix component composition. Using experimental data, we have generated and fitted a model, which we then used to find the local optima for each component in the reaction mix. Using our improved reaction mix composition, we have demonstrated enhanced productivity when synthesizing two model proteins, firefly luciferase and human serum albumin (HSA). Additionally, by generating a statistical model, we have been able to estimate the main effects impacting productivity in the *P. pastoris* cell-free system, which could assist future efforts to engineer and optimise the platform. Increased system understanding could also help to reduce variability for future users by elucidating the sensitivities of the system. By demonstrating improvements to the *P. pastoris* platform and enhanced system understanding using our minimised approach, we hope to make the case that undertaking DOE using cell-free systems need not be limited by access to automation, and that characterisation and optimisation can be achieved without complex liquid-handling platforms.

## 2 Materials & methods

### 2.1 Strains and growth conditions

Plasmid DNA was prepared from *E. coli* strain NEB 5‐α strains (New England Biolabs (NEB), Hertfordshire, UK) cultured in standard Miller lysogeny broth (LB) medium (Sigma-Aldrich, Dorset, UK) supplemented with 37 μg ml^−1^ Kanamycin (Sigma‐Aldrich) for plasmid selection. The *P. pastoris* strain FHL1 was described previously [21] and was cultured in baffled glass flasks in yeast peptone dextrose medium (2% peptone from casein, 1% yeast extract, and 2% dextrose) with 350 μg ml^−1^ Geneticin (VWR, Lutterworth, UK) for selection.

### 2.2 Crude extract preparation and coupled cell-free protein synthesis

*P. pastoris* extracts were prepared as previously described from cells grown at 30 °C, 250 rpm to OD_600_ of 18–20 [21]. Cell-free protein synthesis reactions were performed at 21 °C for 3–6 h with no shaking in a total volume of 50 μL. The reaction mix was varied according to the experimental design as described below, but the ratio of lysate to reaction mix was kept constant at 1:1. Coupled *in vitro* transcription translation reactions were prepared on ice containing: 40 nM DNA, 25 mM HEPES–KOH pH 7.4, 120 mM potassium glutamate, 6 mM magnesium glutamate, 1.5 mM adenosine triphosphate (ATP), 2 mM guanosine triphosphate (GTP), 2 mM cytidine triphosphate (CTP), 2 mM uridine triphosphate (UTP), 0.6 mM of each of 19 amino acids with the exception of leucine at 0.5 mM (biotechrabbit GmbH, Hennigsdorf, Germany), 25 mM creatine phosphate, 2 mM DTT, 0.54 mg ml^−1^ creatine phosphokinase (C3755–1KU, Sigma‐Aldrich), 200 U ml^−1^ RNase Inhibitor (New England Biolabs), 100 U T7 polymerase (Thermo Fisher Scientific). All reaction mix components were thawed on ice fully beforehand prior to being combined. Additionally, all reaction components were added in the sequence described, in a single 1.5 mL Eppendorf tube (Eppendorf, Hamburg, Germany) per reaction. For the DoE experiments, cell-free reaction mix components were combined into 21 separate master mix solutions on ice, with only two master mix solutions being prepared and used at a time to ensure minimal wait times between addition of components.

### 2.3 Luciferase assay

In all cases, wild-type firefly luciferase derived from *Photinus pyralis* was used as a reporter protein. The amino acid sequence for firefly luciferase used in this study is available in the Supplementary Information. A luciferase assay mix was formulated consisting of 20 mM tricine, 2.67 mM MgSO_4,_ 1.07 mM MgCO_3_, 100 μM EDTA and 17 mM DTT along with 250 μM luciferin and 250 μM ATP [33], which was freshly prepared before each assay. 5 μl of the cell‐free reaction was added to 30 μl of the luciferase assay mix every 30 min, and the luminescence was measured using the CLARIOstar Plus ® Omega plate reader (BMG Labtech Ltd, Aylesbury, UK). Average readings were taken over 20 min as reported previously [15,21].

### 2.4 Human serum albumin assay (HSA)

HSA was quantified using the Albumin Blue Fluorescent assay kit (Active Motif, La Hulpe, Belgium) according to the manufacturer's protocol. The amino acid sequence for HSA used in this study is available in the Supplementary Information.

### 2.5 Design of experiments (DOE)

The 7 components included in the DOE as factors were selected based on initial screening experiments, those previously highlighted as important in the literature, and those which could be easily modulated. The design itself was a Definitive Screening Design (DSD), and all factors were three-level, numeric and continuous. The option to add blocks was selected with centre points to estimate quadratic effects. The 22 runs were split into 2 blocks with 4 extra runs, 2 runs of which were centre points. Run order was randomized within the blocks. The attribute selected for the response was luminescence (AU) as a proxy for luciferase yield. JMP Pro 14 (SAS Institute Inc., Cary, USA) was used to generate a randomized 7-factor DOE consisting of 22 runs and 2 centre-points.

## 3 Results and discussion

### 3.1 Screening components to identify relevant factors

The three main components of a CFPS reaction are the cellular extract, genetic template and the reaction mixture, formulated to sustain transcription and translation. The *P. pastoris* CFPS system previously developed had undergone optimisation to improve productivity focusing on two of the three main components. The cellular extract had been improved by developing lysate preparation methodologies and by generation of a ribosome biogenesis-modified strain from which the lysate is derived. With respect to the genetic template, incorporation of an internal ribosome entry site (IRES) element originating from the cricket paralysis virus (CrPV) into the template design led to an increase in product yield [21]. As previous improvements to the system focused solely on the cellular extract and genetic template, we turned our attention towards the composition of the reaction mix. The importance of the reaction mix, both with respect to the productivity and overall robustness of the system, has been demonstrated [34,35]. CFPS systems have multiple potential applications and each system has distinct advantages and disadvantages. Therefore, what is considered improvement for one system or application might not necessarily translate directly to another application or system. In our case, we aimed to maximise productivity, defined as the amount of functional protein expressed in our system. Therefore, one of the key objectives in this study was to determine what components in the reaction mix are limiting factors in protein synthesis yield. The second goal of this study was to improve the robustness of the system to overcome slight variances in reaction set-up, and therefore output, with respect to productivity. By employing a DOE approach, we hoped to glean information related to the sensitivities of the system so that components contributing to variance could be monitored and restricted more rigorously in future. Using this approach could therefore enable simpler technology transfer and adoption for new users. It is common knowledge that variability between laboratories and users exists, even when exchanging the same materials and personnel [34]. Understanding and limiting these underlying sources of variation is a key hurdle to overcome prior to widespread deployment of CFPS as the field shifts towards more application-focused research.

To this end, we initially sought to identify factors to be included in the design. Our chosen factors were selected primarily from literature evidence, where similar optimisation of the reaction mix had been conducted, albeit in different cell-free systems [30,32]. Next, using firefly luciferase as a reporter protein, we initially screened these components individually at three different concentrations. By conducting these initial screens, we aimed to ascertain the edges of the design space to provide an indication whether the optima fell within our selected range and to exclude component concentrations that result in failed reactions, which compromises the overall design. We also wanted to eliminate factors with unwanted effects and those which had no observable impact on the expression of luciferase to allow a more detailed investigation of the remaining factors. Finally, we needed to independently verify the impact of changing the concentration of Mg^2+^ and ATP on the luciferase assay, as these components would be present in the design at different concentrations and therefore might impact our estimations of productivity. All reactions were conducted in 50 μL static reactions for 5 h at 21 °C.

**Fig. 1:**
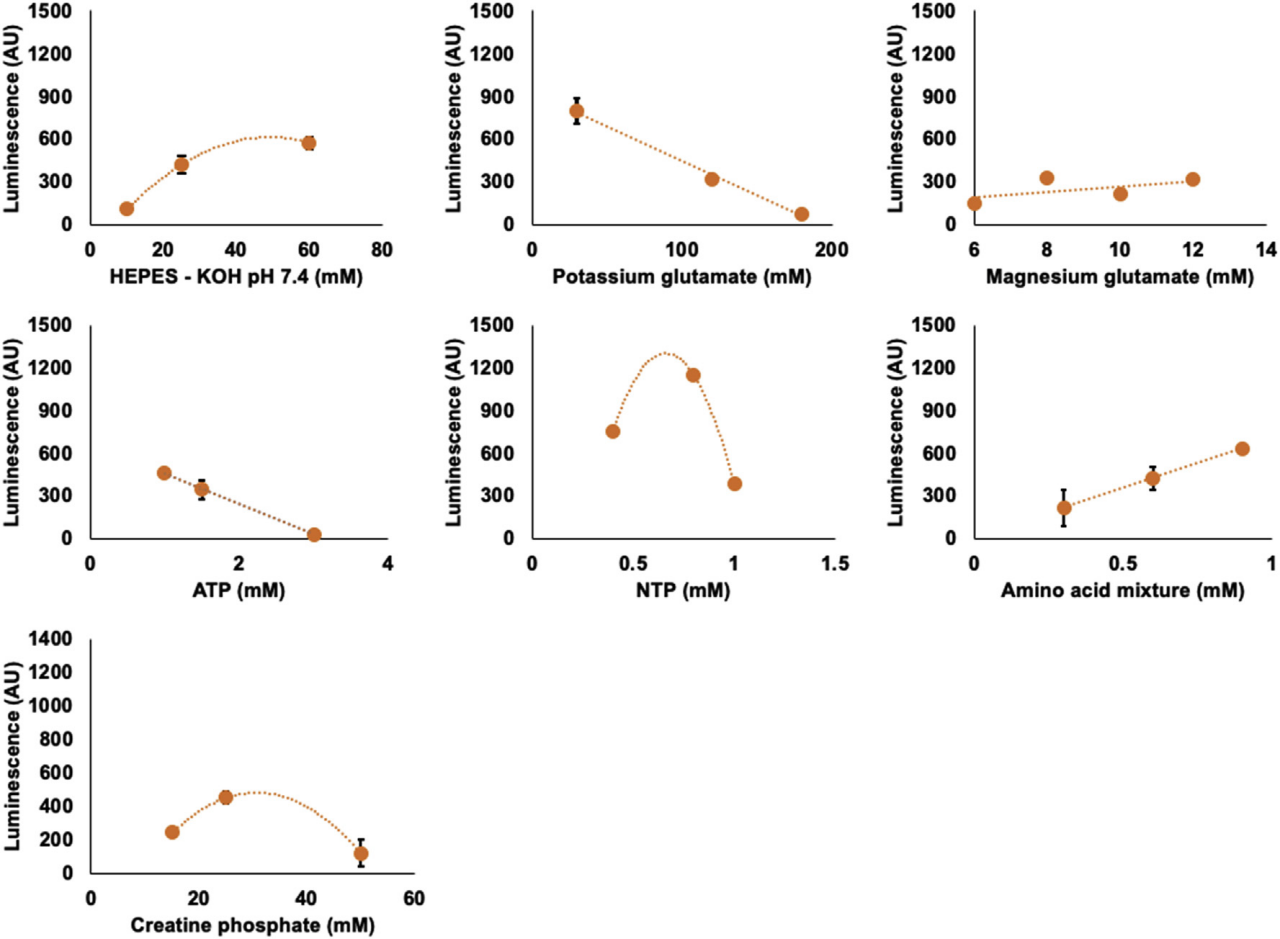
Initial screening experiments assessing individual component concentrations on the production of luciferase. For each selected concentration, luminescence was read hourly for 6 h and an average taken once luminescence had plateaued. Line of fit displays either linear or quadratic fitting of the datapoints.

Based on the initial screening trials, it was found that altering the concentrations of DTT and creatine kinase did not affect synthesis yield significantly (Supplementary Fig. 1) and therefore these were left unaltered in the DOE design except for the concentration of creatine kinase which was reduced from 0.54 mg ml^−1^ to 0.27 mg ml^−1^ in order to improve the cost-effectiveness of the system. Similar observations were reported when optimising the reaction mix of an *E. coli* cell-free system [32]. Altering the concentration of magnesium glutamate seemingly had little impact on the expression of luciferase (Fig. 1), which was somewhat surprising given that Mg^2+^ was noted in early studies to be one of the first critical component concentrations to be optimised for each batch of extract to improve productivity [36]. Additionally, increasing the concentration of ATP in the reaction mix resulted in a decrease in the expression of luciferase (Fig. 1). These results also show that increasing the concentration of ATP and Mg^2+^ in the reaction mix has no positive impact on the luciferase assay, suggesting these two compounds are not rate limiting in the photon generation reaction. However, increasing the concentration of ATP seemingly had a negative impact on the productivity of the cell-free system, leading to reduced protein synthesis. This could potentially be the result of increased accumulation of inorganic phosphate as a result of ATP degradation, which inhibits translation by binding and sequestering Mg^2+^ which is essential for protein synthesis and other nucleotide-dependent reactions [37,38].

### 3.2 Improving the CFPS reaction mix composition

Based on the initial screening experiments, seven factors were selected to be included in the design: HEPES-KOH buffer, potassium glutamate, magnesium glutamate, ATP, NTP mixture (CTP/GTP/UTP), creatine phosphate and the amino acid mixture. Even though it showed little effect in the initial screen, we decided to include magnesium glutamate in the design given it is involved in many steps of transcription and translation, such as aminoacylation of tRNA [39], coordination of NTPs in the active site of the RNA polymerase [40] and stability of ribosomes is highly dependent on Mg^2+^ concentration [41,42]. Based on the known importance of Mg^2+^ ions, it is likely there would be secondary interactions with other factors which could affect protein synthesis that were unable to be elucidated using the one-factor-at-a-time approach employed in the initial screening experiments. The component concentration range of creatine phosphate was also altered from the initial screen (from 50 mM to 45 mM) in order to reduce the possibility of a failed reaction that would compromise the overall design. High initial concentrations of creatine phosphate likely result in increased inorganic phosphate present in the reaction environment, thereby inhibiting protein synthesis, similar to the effect observed with increased ATP concentrations. With respect to potassium glutamate and ATP, the initial screens indicated a positive effect on protein synthesis when the concentration of these components was lowered. As such, the lower component concentration level was included as 0 mM for potassium glutamate and ATP. Here the component volume was substituted with ddH_2_O.

In the past, screening experiments were typically conducted using resolution III and IV DOE designs to elucidate significant parameters or factors. However, one of the detriments of using resolution III designs is the confounding of main effects with two-factor interactions, should they be active [43]. Additionally, when using resolution IV designs, two-factor interactions are confounded with other two-factor interactions. Definitive Screening Designs (DSD) were developed to allow main effects and interactions to be determined for multiple factors using correlation-optimised designs [43]. Some of the advantages of using a DSD over traditional screening designs is that main effects are orthogonal and entirely free of aliasing with two-factor and quadratic interactions. Additionally, DSDs can elucidate main effects with symmetric curvature [43]. A further advantage, and one of the key motivations for selecting a DSD in this study, is that far fewer runs are required for DSDs than fractional factorial designs [44].

Selected factors were screened using a DSD design consisting of 22 runs and two centre-points (Supplementary Information, Table 1). The option to add blocks was selected with centre points to estimate quadratic effects. The 22 runs were split into 2 blocks with 4 extra runs, 2 runs of which were centre points. Run order was randomized within the blocks. The design was selected to reveal the main effects on protein synthesis in the system to identify the optimum component concentrations in a minimum number of runs whilst also identifying key components contributing to variance. Table 2 shows the selected concentration ranges for each of the included factors.

**Table 1:** Components included in the design as factors and their respective concentrations (mM) at 3-levels.

	HEPES KOH pH 7.4	Potassium glutamate	Magnesium glutamate	ATP	NTPs	Creatine phosphate	Amino acids^a^
**Low**	10	0	6	0	0.4	15	0.3
**Medium**	35	70	10	1	0.7	30	0.6
**High**	60	140	14	2	1	45	0.9

a Of 19 amino acids, 0.25–0.5-0.75 of Leucine.

**Table 2:** Low, middle and high concentration levels of factors screened in the experimental design.

Factor	Component	Screened levels (mM)
A	HEPES-KOH, pH 7.4	10-35-60
B	Potassium glutamate	0-70-140
C	Magnesium glutamate	6-10-14
D	ATP	0-1-2
E	NTP mixture (CTP/GTP/UTP)	0.4–0.7-1
F	Creatine phosphate	15-30-45
G	Amino acid mixture	0.3–0.6-0.9^a^

a Of 19 amino acids, 0.25–0.5-0.75 of Leucine.

**Fig. 2:**
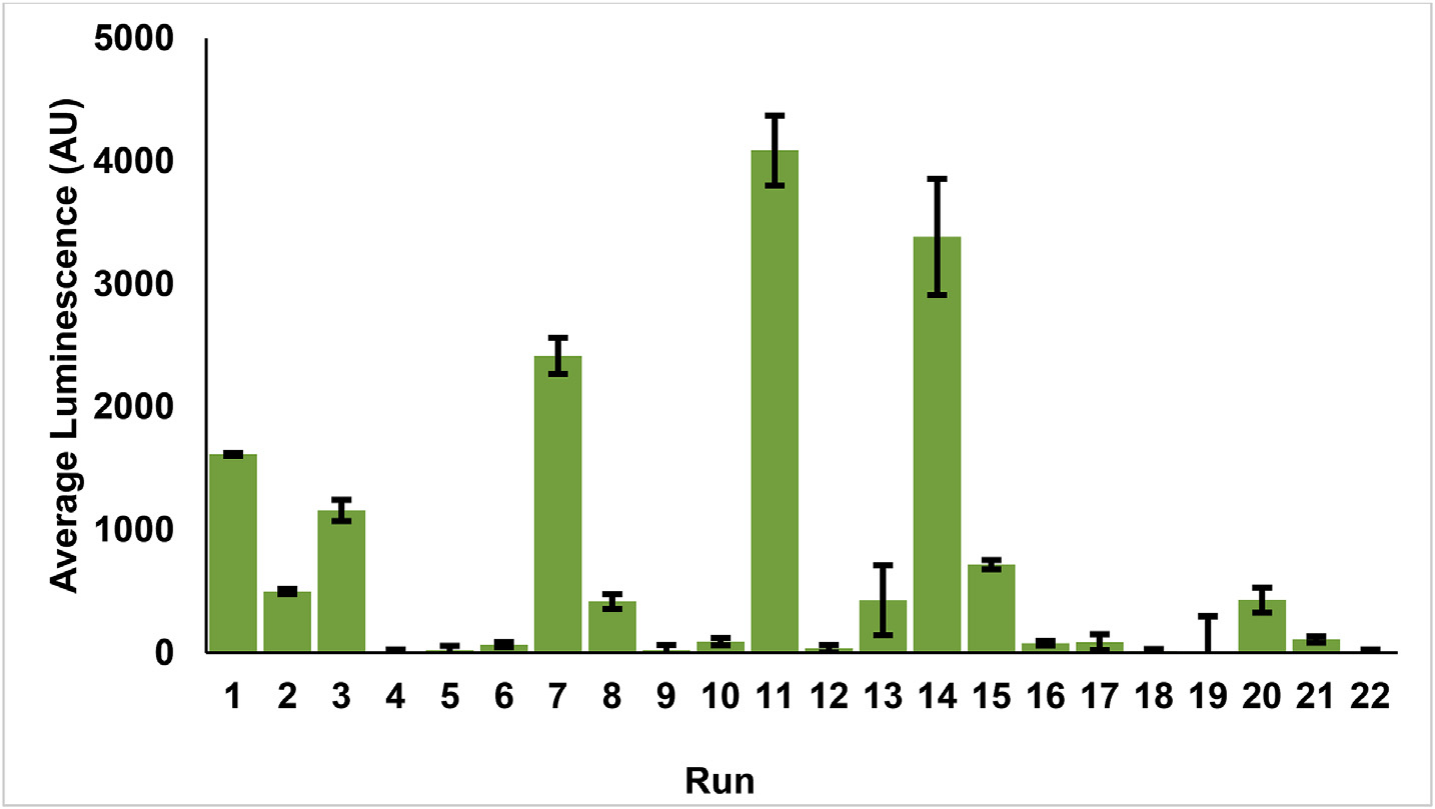
All experimental runs from the 22-run design. Luciferase production was measured over 4 h, with samples taken every hour and the average taken from three technical replicates after the luminescence value had plateaued. The error bars indicate the standard deviation of three technical repeats and are calculated using error propagation. Experimental conditions for each of the 22 runs can be found in the Supplementary Information, Table 1.

The expression of luciferase for each run was measured and an average of the arbitrary luminescence units (AU) taken after the luminescence signal had plateaued. Negative control reactions were also set up for each tested reaction condition. Negative control reactions contained all components at the concentrations present in the corresponding run aside from the DNA template, where the volume was substituted with deionised water. The average luminescence signal from each negative control reaction was used to subtract background luminescence signal from the corresponding run. The results of these runs can be observed in Fig. 2. The average luminescence value for each of the runs was then used to fit the model. Runs that did not produce any measurable outcome were excluded from further statistical analysis and fitting of the model.

Stepwise linear regression using the minimum corrected Akaike Information Criterion was utilised to determine the best model (fit least squares) and the factors to be included therein, with effects entering the model in the forward direction. The ANOVA table showed that the model was significant (*P* = 0.0192), however the fitness of the model was shown to be fairly poor with R^2^ and adjusted R^2^ values of 0.61 and 0.46, respectively. Despite this, strong effects on yield were observed with HEPES, potassium glutamate and creatine phosphate (Supplementary Information, Table 2).

Using the model and estimates of the main effects, we were able to determine concentrations of components in the reaction mix that enhance productivity in the system. The improved reaction mix is summarised in Table 3 and shown alongside previously established conditions [21]. It is worth noting that these improved component concentrations do not take into consideration the amounts already present in the lysate. However, we expect these to be minimal due to the dialysis process during lysate preparation, aside from potassium glutamate, which is present in the dialysis buffer (120 mM potassium glutamate).

**Table 3:** Established and improved concentrations of reaction components in the coupled *Pichia pastoris* CFPS system.

Component	Established concentration	Improved concentration
HEPES KOH pH 7.4	25 mM	60 mM
K-glutamate	120 mM	0 mM
Mg-glutamate	6 mM	6 mM
DTT	2 mM	2 mM
Amino acids mixture	0.6 mM each 19 amino acids, 0.5 mM Leucine (RTS amino acids mix)	0.9 mM each 19 amino acids, 0.75 mM Leucine (RTS amino acids mix)
ATP	1.5 mM	1 mM
NTP mixture (CTP/GTP/UTP)	1 mM	1 mM
Creatine phosphate	25 mM	45 mM
Creatine phosphokinase	0.54 mgml^−1^	0.27 mgml^−1^
RNase Inhibitor	200 U/ml	200 U/ml
T7 RNA polymerase	100 U	100 U
DNA Template	40 nM	40 nM

The importance of ATP and creatine phosphate in the reaction mix are inextricably linked, as it is well known that a stable supply of ATP is one of the key factors affecting the efficiency and success of CFPS [45,46]. ATP plays critical roles in multiple steps from genetic template to protein translation and therefore a reasonable and constant supply is required for prolonged reaction longevity. ATP is regenerated in our reaction mix by substrate-level phosphorylation of ADP using high-energy phosphate bonds. In this system creatine phosphate is used as a high-energy phosphate molecule, which when used in conjunction with creatine kinase regenerates ATP to power the reaction. As is the issue with other phosphate-containing molecules used to power CFPS, the accumulation of inorganic phosphate unavoidably occurs, leading to sequestration of magnesium ions in the reaction mixture reducing protein synthesis [38].

Paired ionic compounds such as potassium glutamate make a key contribution to the overall ionic strength of the reaction environment, this in turn modulates the electrostatic interactions of proteins [47,48]. This delicate balance can have implications on enzyme activity [49], protein aggregation [50], protein solubility [51] and protein-nucleic acid interactions [52]. Often the ionic strength in CFPS is tied to the extract itself and must be altered between batches in order to find the optimum. The potassium cation (K^+^) in high enough concentrations can lead to dissociation of ribosomes *in vitro* [53,54] due to competitive binding with Mg^2+^ [54,55]. It is also hypothesised that after cell lysis, the overall concentration of K^+^ drops, and it is therefore essential to include it in the reaction mix in order to restore ionic homeostasis. It is interesting therefore to note that the optimum concentration suggested by the model in the reaction mix is 0 mM potassium glutamate. This does not consider the amounts of potassium glutamate which may carry over from the lysis and dialysis buffers (120 mM potassium glutamate). Despite no additional supplementation when formulating the reaction mix, the concentration of potassium glutamate is clearly sufficient for protein synthesis to occur. Further efforts to optimise potassium glutamate concentration could look at optimising the concentration added during lysate preparation.

During growth, cells are able to maintain their cytoplasmic pH through various mechanisms, however these are thought to be interrupted to the degree where they are no longer functional in cell-free lysates due to significant disruption to the genome and cellular membrane [35]. Maintaining the pH and ionic strength in cell-free lysates is therefore achieved using a buffering system. HEPES is a very common buffer used in CFPS and compatible with most energy systems, whereby the desired pH range is achieved through titration with KOH usually within the range of pH 6.8 to 8.2 [35]. It has been shown that decreasing pH (pH < 6.2) can have negative effects on expression levels in an *E. coli* cell-free system [56] and that pH can have a significant effect on protein yield and enzyme activity [35,57]. Whilst our experimental design did not include the pH of the HEPES buffer, only the concentration of the buffer in the reaction mix, our results indicate decreasing the concentration of HEPES results in decreased expression levels likely through reduced capacity to maintain an approximation of physiological pH.

Although the amino acid mixture was not identified as a significant main effect in the model, an amino acid mixture is included in all cell-free systems, and it is perhaps unsurprising that an elevated supply of amino acids enabled an increase in protein synthesis. Although we included the amino acid mixture in the design as an individual factor, it is highly likely that it could be only a small subset of the amino acid mixture which is limiting protein synthesis and furthermore this could be protein specific. Further attempts to optimise the system could focus on examining each individual amino acid contribution to the yield of individual proteins, and the individual interactions of specific amino acids with components in the reaction mix. Specific increases in individual amino acids in the mixture have previously been shown to significantly increase protein expression in an *E. coli* CFPS system [32].

### 3.3 Evaluating an improved reaction mix composition on the productivity of the *P. pastoris* CFPS system

**Fig. 3:**
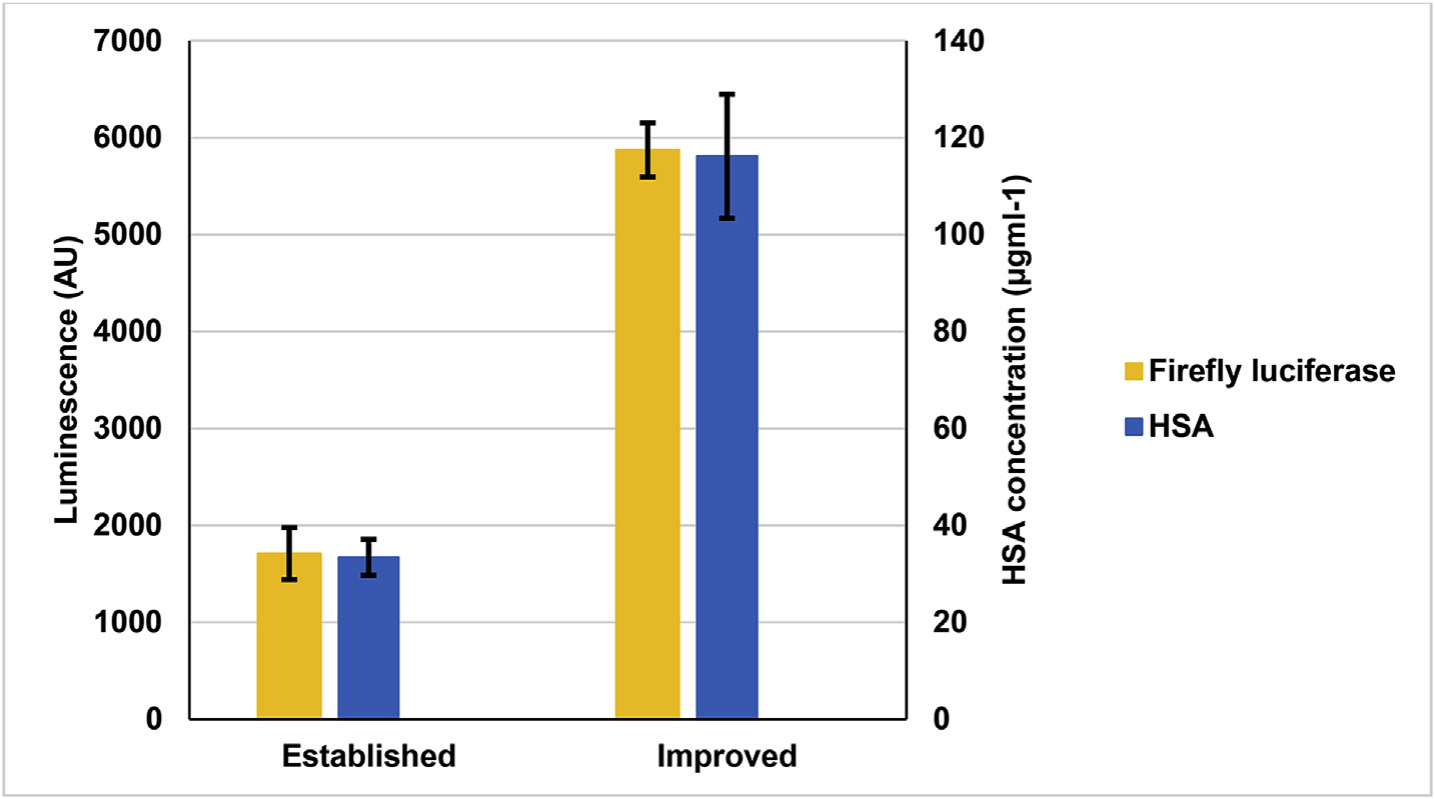
Comparison of CFPS yields using the established and improved reaction mix composition when producing luciferase and HSA. Luciferase production was measured over 4 h, with samples taken every hour. HSA was produced overnight and quantified using the Albumin Blue (AB) fluorescence assay kit. The error bars indicate the standard deviation of three biological repeats and are calculated using error propagation.

To validate the improved conditions as informed by the model, the production of luciferase was repeated using the suggested concentrations for the components indicated to be important in the model (HEPES, potassium glutamate, creatine phosphate, ATP and the amino acid mixture). Other components in the reaction mix not identified as important by the model were maintained at the same concentrations as the previously established reaction mix (Table 3). The production of luciferase using the improved reaction mixture significantly outperformed the predicted relative luminescence signal in the model by approximately 3-fold, with a 4.8-fold increase in relative luminescence signal compared to the previously established conditions (Fig. 3).

Next, we sought to ascertain whether the modifications to the reaction mix enhancing productivity were protein specific. To examine this, we synthesised a more complex protein of biopharmaceutical interest, human serum albumin (HSA), which had also previously been produced in this system [21]. Reactions were conducted in 50 μL at 21 °C without shaking and were run overnight in order to allow sufficient time for disulphide-bond formation. The Albumin Blue (AB) fluorescence assay was used to quantify production of HSA. Using the improved reaction mix composition resulted in a 3.5-fold increase in HSA synthesis compared to the established reaction mix composition (116.2 μg ml^−1^ and 33.4 μg ml^−1^, respectively. Fig. 3).

Based on these findings, we conclude that the improved reaction mix is not entirely protein specific, due to the increases in both luciferase and HSA production, and the fact that the fold-change is relatively consistent between the two proteins. The slight disparity in fold-change between luciferase and HSA production when comparing established and optimised reaction mixes could be accounted for by the relative complexity of HSA, given that the protein contains 17 disulphide-bridges [58] and the Albumin Blue (AB) fluorescence assay only produces a fluorescence signal should the lipid binding pocket be fully formed. It is possible therefore that the disparity in fold-change could be accounted for by a proportion of the HSA protein having incomplete or incorrect lipid binding pocket formation. Despite this, the yield of HSA produced in our system is still higher than previously reported yields of GFP in HeLa [8] and RRL [59] systems, and luciferase in CHO [14], insect [60] and *S. cerevisiae* [15] systems.

## 4 Conclusion

The aim of this work was to improve a *P. pastoris*-based cell-free platform using a minimised DOE approach, focusing solely on the reaction mix composition. In a limited number of experiments, and without the use of automation, we were able to elucidate component concentrations enhancing productivity of the system and estimate the main effects. As a result, we achieved a 4.8-fold increase in relative luminescence signal compared to established reaction mix conditions and a 3.5-fold increase in synthesis of the complex biopharmaceutical HSA. As a consequence, we have demonstrated that optimisation and characterisation of cell-free systems need not be conducted by laborious and low-resolution one-factor-at-a-time approaches or be limited by access to liquid handling automation.

This work further establishes the *P. pastoris* platform as a highly productive and versatile eukaryotic cell-free system. Having previously demonstrated the ability to produce two complex protein classes: proteins containing disulphide bonds [21] and virus-like particles (VLPs) [22], we hope to further expand the potential scope of the platform by demonstrating synthesis of diverse protein classes. In doing so, the authors anticipate utilisation of the system as a highly adaptable prototyping platform to enhance biopharmaceutical research *in vivo*.

## Author contributions statement

AS designed and performed the experiments. RA and KP helped design the experiments. KP and DB helped to conceive the study. All authors helped draft the manuscript and read and approved the final manuscript.

## CRediT authorship contribution statement

**Alex J. Spice:** Conceptualization, Data curation, Formal analysis, Investigation, Methodology, Writing - original draft. **Rochelle Aw:** Conceptualization, Supervision, Writing - review & editing. **Daniel G. Bracewell:** Conceptualization, Funding acquisition, Writing - review & editing. **Karen M. Polizzi:** Conceptualization, Funding acquisition, Supervision, Writing - review & editing.

## Declaration of competing interest

The authors declare no competing interests.
