# Supplementary material for: Improving the reaction mix of a Pichia pastoris cell-free system using a design of experiments approach to minimise experimental effort
Source: Synth Syst Biotechnol. 2020 Jun 23;5(3):137–44. doi: 10.1016/j.synbio.2020.06.003 (PMC7320237; doi:10.1016/j.synbio.2020.06.003)
Supplement: Multimedia component 1 [file mmc1.docx]

**Supplementary Information**

**
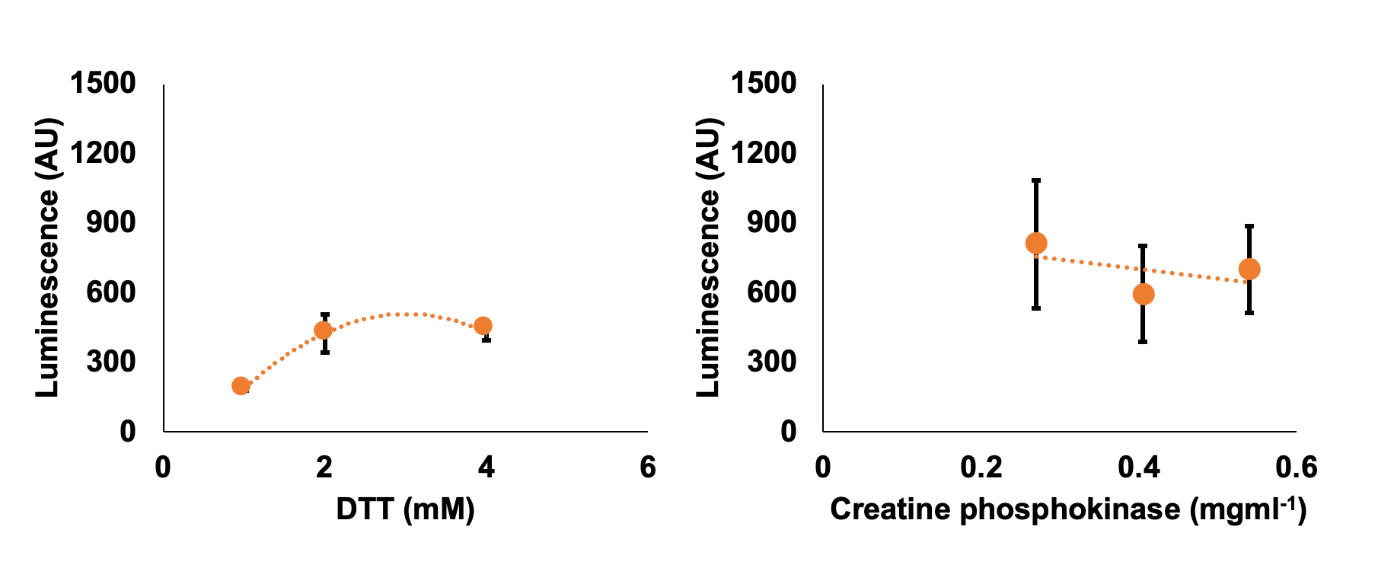
**

**Figure 1. Initial screening experiments assessing the impact of DTT and creatine phosphokinase concentrations on the production of luciferase. For each selected concentration, luminescence was read hourly for 6 hr and an average taken once luminescence had plateaued. Line of fit displays either linear or polynomial curve fitting of the datapoints.**

**Table 1: Experimental conditions for each run in the DSD experimental design. All concentrations are in mM.**

| Run | Block | HEPES | K-glutamate | Mg-glutamate | ATP | NTPs | Creatine phosphate | Amino acids |
| --- | --- | --- | --- | --- | --- | --- | --- | --- |
| 1 | 1 | 60 | 0 | 14 | 2 | 0.7 | 15 | 0.3 |
| 2 | 1 | 10 | 0 | 6 | 2 | 1 | 15 | 0.9 |
| 3 | 1 | 35 | 140 | 14 | 2 | 1 | 45 | 0.9 |
| 4 | 1 | 35 | 0 | 6 | 0 | 0.4 | 15 | 0.3 |
| 5 | 1 | 10 | 140 | 6 | 0 | 0.7 | 45 | 0.9 |
| 6 | 1 | 10 | 140 | 10 | 2 | 0.4 | 15 | 0.9 |
| 7 | 1 | 60 | 0 | 10 | 0 | 1 | 45 | 0.3 |
| 8 | 1 | 35 | 70 | 10 | 1 | 0.7 | 30 | 0.6 |
| 9 | 1 | 60 | 140 | 14 | 0 | 0.4 | 45 | 0.3 |
| 10 | 1 | 60 | 140 | 6 | 2 | 0.4 | 45 | 0.6 |
| 11 | 1 | 10 | 0 | 14 | 0 | 1 | 15 | 0.6 |
| 12 | 2 | 60 | 0 | 6 | 1 | 1 | 45 | 0.9 |
| 13 | 2 | 10 | 0 | 14 | 0 | 0.4 | 45 | 0.9 |
| 14 | 2 | 60 | 0 | 14 | 2 | 0.4 | 30 | 0.9 |
| 15 | 2 | 10 | 70 | 14 | 2 | 1 | 45 | 0.3 |
| 16 | 2 | 60 | 140 | 6 | 2 | 1 | 15 | 0.3 |
| 17 | 2 | 10 | 140 | 6 | 0 | 1 | 30 | 0.3 |
| 18 | 2 | 60 | 140 | 14 | 0 | 1 | 15 | 0.9 |
| 19 | 2 | 10 | 140 | 14 | 1 | 0.4 | 15 | 0.3 |
| 20 | 2 | 35 | 70 | 10 | 1 | 0.7 | 30 | 0.6 |
| 21 | 2 | 10 | 0 | 6 | 2 | 0.4 | 45 | 0.3 |
| 22 | 2 | 60 | 70 | 6 | 0 | 0.4 | 15 | 0.9 |

**Table 2: Parameter estimates for important factors in the DSD design and pareto plot**

| **Term** | **t Ratio** | **Prob>\|t\|** |
| --- | --- | --- |
| HEPES (10,60) | 2.73 | 0.0173* |
| K glutamate (0,140) | -2.56 | 0.0239* |
| ATP (0,2) | 0.42 | 0.6828 |
| Creatine phosphate (15,45) | 2.21 | 0.0454* |
| Amino acids (0.3,0.9) | 1.98 | 0.0695 |

Amino acids sequences for reporter proteins used in this study:

Firefly Luciferase

>tr|Q27758|Q27758_PHOPY Firefly luciferase OS=Photinus pyralis OX=7054 GN=luc PE=4 SV=1

MEDAKNIKKGPAPFYPLEDGTAGEQLHKAMKRYALVPGTIAFTDAHIEVNITYAEYFEMS

VRLAEAMKRYGLNTNHRIVVCSENSLQFFMPVLGALFIGVAVAPANDIYNERELLNSMNI

SQPTVVFVSKKGLQKILNVQKKLPIIQKIIIMDSKTDYQGFQSMYTFVTSHLPPGFNEYD

FVPESFDRDKTIALIMNSSGSTGSPKGVALPHRTACVRFSHARDPIFGNQIIPDTAILSV

VPFHHGFGMFTTLGYLICGFRVVLMYRFEEELFLRSLQDYKIQSALLVPTLFSFFAKSTL

IDKYDLSNLHEIASGGAPLSKEVGEAVAKRFHLPGIRQGYGLTETTSAILITPEGDDKPG

AVGKVVPFFEAKVVDLDTGKTLGVNQRGELCVRGPMIMSGYVNDPEATNALIDKDGWLHS

GDIAYWDEDEHFFIVDRLKSLIKYKGCQVAPAELESILLQHPNIFDAGVAGLPGDDAGEL

PAAVVVLEHGKTMTEKEIVDYVASQVTTAKKLRGGVVFVDEVPKGLTGKLDARKIREILI

KAKKGGKSKL

Human Serum Albumin (HSA)

>CAA23754.1:19-609 serum albumin [Homo sapiens]

MRGVFRRDAHKSEVAHRFKDLGEENFKALVLIAFAQYLQQCPFEDHVKLVNEVTEFAKTCVADESAENCDKSLHTLFGDKLCTVATLRETYGEMADCCAKQEPERNECFLQHKDDNPNLPRLVRPEVDVMCTAFHDNEETFLKKYLYEIARRHPYFYAPELLFFAKRYKAAFTECCQAADKAACLLPKLDELRDEGKASSAKQRLKCASLQKFGERAFKAWAVARLSQRFPKAEFAEVSKLVTDLTKVHTECCHGDLLECADDRADLAKYICENQDSISSKLKECCEKPLLEKSHCIAEVENDEMPADLPSLAADFVESKDVCKNYAEAKDVFLGMFLYEYARRHPDYSVVLLLRLAKTYETTLEKCCAAADPHECYAKVFDEFKPLVEEPQNLIKQNCEL

FKQLGEYKFQNALLVRYTKKVPQVSTPTLVEVSRNLGKVGSKCCKHPEAKRMPCAEDYLSVVLNQLCVLHEKTPVSDRVTKCCTESLVNRRPCFSALEVDETYVPKEFNAETFTFHADICTLSEKERQIKKQTALVELVKHKPKATKEQLKAVMDDFAAFVEKCCKADDKETCFAEEGKKLVAASQAALGL
